# Identification and functional characterization of BICD2 as a candidate disease gene in an consanguineous family with dilated cardiomyopathy

**DOI:** 10.1186/s12920-022-01349-y

**Published:** 2022-09-06

**Authors:** Kai Luo, Chenqing Zheng, Rong Luo, Xin Cao, Huajun Sun, Huihui Ma, Jichang Huang, Xu Yang, Xiushan Wu, Xiaoping Li

**Affiliations:** 1 Department of Cardiology, Sichuan Provincial People’s Hospital, University of Electronic Science and Technology of China, Chengdu, 610072 Sichuan People’s Republic of China; 2 Chinese Academy of Sciences Sichuan Translational Medicine Research Hospital, Chengdu, 610072 People’s Republic of China; 3 Shenzhen Aone Medical Laboratory Co., Ltd., Shenzhen, People’s Republic of China; 4 Institute of Geriatric Cardiovascular Disease, Chengdu Medical College, Chengdu, People’s Republic of China; 5 School of Acupuncture-Moxibustion and Tuina, Chengdu University of Traditional Chinese Medicine, Chengdu, People’s Republic of China; 6 Department of Pathology, Sichuan Provincial People’s Hospital, University of Electronic Science and Technology of China, Chengdu, 610072 Sichuan People’s Republic of China; 7 The Center for Heart Development, Hunan Normal University, Changsha, People’s Republic of China; 8 Guangdong Provincial Key Laboratory of Pathogenesis, Targeted Prevention and Treatment of Heart Disease, Guangzhou, Guangdong People’s Republic of China

**Keywords:** Dilated cardiomyopathy, *BICD2*, Zebrafish model, RNA-seq

## Abstract

**Background:**

Familial dilated cardiomyopathy (DCM) is a genetic cardiomyopathy that is associated with reduced left ventricle function or systolic function. Fifty-one DCM-causative genes have been reported, most of which are inherited in an autosomal dominant manner. However, recessive DCM-causative gene is rarely observed.

**Methods:**

Whole-exome sequencing (WES) was performed in a consanguineous family with DCM to identify candidate variants. Sanger sequencing was utilized to confirm the variant. We then checked the DCM candidate gene in 210 sporadic DCM cases. We next explored *BICD2* function in both embryonic and adult *bicd2*-knockout zebrafish models. In vivo cardiac function of *bicd2*-knockout fish was detected by echocardiography and RNA-seq.

**Results:**

We identified an autosomal recessive and evolutionarily conserved missense variant, NM_001003800.1:c.2429G > A, in *BICD2*, which segregated with the disease phenotype in a consanguineous family with DCM. Furthermore, we confirmed the presence of *BICD2* variants in 3 sporadic cases. Knockout of *bicd2* resulted in partial embryonic lethality in homozygotes, suggesting a vital role for *bicd2* in embryogenesis. Heart dilation and decreased ejection fraction, cardiac output and stroke volume were observed in *bicd2*-knockout zebrafish, suggesting a phenotype similar to human DCM. Furthermore, RNA-seq confirmed a larger transcriptome shift in in *bicd2* homozygotes than in heterozygotes. Gene set enrichment analysis of *bicd2*-deficient fish showed the enrichment of altered gene expression in cardiac pathways and mitochondrial energy metabolism.

**Conclusions:**

Our study first shows that BICD2 is a novel candidate gene associated with familial DCM, and our findings will facilitate further insights into the molecular pathological mechanisms of DCM.

**Supplementary Information:**

The online version contains supplementary material available at 10.1186/s12920-022-01349-y.

## Introduction

Dilated cardiomyopathy (DCM) is one of the major cardiomyopathy subtypes [1]. It is the third leading cause of heart failure and the most common cause of heart transplantation [2], but its aetiology and pathogenesis remain elusive.

Familial DCM is now more commonly diagnosed owing to the improved awareness of disease-causing genetic variants and genetic screening. Familial DCM is found in 20–35% of patients with DCM, 80% of DCM patients inherit in autosomal dominant pattern, and 10–15% of DCM patients exhibit autosomal repressive or X-linked inheritance [3]. Previous DCM studies revealed > 250 associated genes spanning > 10 gene ontologies, suggesting a complex and diverse genetic architecture [4]. A DCM gene curation expert panel curated a final set of 51 genes proposed to have a monogenic role in isolated, idiopathic DCM in humans [4]. In addition, 12 out of these 51 genes, namely, *BAG3*, *DES*, *FLNC*, *LMNA*, *MYH7*, *PLN*, *RBM20*, *SCN5A*, *TNNC1*, *TNNT2*, *TTN*, and *DSP*, were classified as having definitive or strong evidence [4]. Another 7 genes, *ACTC1*, *ACTN2*, *JPH2*, *NEXN*, *TNNI3*, *TPM1*, and *VCL,* were classified as having moderate evidence [4]. However, these 19 genes explain only a minority of cases, leaving the majority of the genetic architecture of DCM incompletely addressed [4].

Most reported DCM variants are inherited in an autosomal dominant manner [3–5]; autosomal recessive (AR) inheritance in adult DCM is less frequently observed [6] and is more common in paediatric DCM [7–9]. Consanguineous families are ideal for investigating AR DCM. However, few studies have focused on familial DCM in consanguineous families [9–11].

Bicaudal-D2 (*BICD2*) is a dynein-activating adaptor protein that plays a critical role in minus-end-directed microtubule-based transport [12, 13]. Mutations in human *BICD2* have been linked to a spectrum of neuronal disorders and particularly to a dominant mild early onset form of spinal muscular atrophy [14–17]. The *BICD2* protein could bind to the kinesin tail and kinesin-activating protein filaments, which then activates the kinesin and allows the kinesin complex to travel along the microtubule cytoskeleton to transport cargo [18]. *Bicd2*-KO mice showed significantly lower *LMNA* expression at the protein level, decreased *MKL-1*/*SRF* activity, and significantly downregulated expression of the downstream α-actin, α-coactin and membrane association proteins, as well as microtubulin [19]. *MKL-1*, a member of the actin family, plays an important role in cardiovascular system development and functions as a cofactor of serum response factor (*SRF*), which activates *SRF*-dependent transcriptional regulatory elements. In nuclear fibrillar protein A/C gene (*LMNA*) knockout mice, the *MKL1*/*SRF* signalling pathway is also inhibited, and the nuclear translocation of endogenous *MKL-1* in cardiomyocytes is dysfunctional, leading to reduced expression of *SRF*, α-actinin and membrane association proteins and subsequently the development of dilated heart disease [20]. Simple blockade of *SRF* expression in mouse myocardial tissue can lead to the development of DCM [21].

The zebrafish model of DCM is one of the ideal systems in which to study the function of gene mutations in DCM. These model zebrafish harbour an enlarged heart, reduced shortening fraction during cardiac contraction and no cardiomyocyte proliferation, consistent with the characteristics of human DCM [22].

In this study, we performed whole-exome sequencing (WES) in a consanguineous family with DCM to identify candidate DCM-causing genes. Subsequently, functional studies of the candidate DCM gene *BICD2* were conducted in zebrafish to demonstrate the relationship between *BICD2* and DCM.

## Materials and methods

### Patients and clinical evaluation

The proband and his families from consanguineous family was recruited in 2011. In total, 210 sporadic DCM patients were enrolled for further *BICD2* variant validation. Written informed consent was obtained from all subjects participating.

### Genomic DNA preparation

Peripheral blood from DCM cases was collected into EDTA anticoagulant tubes, and genomic DNA was extracted using a blood DNA extraction kit according to the manufacturer’s protocol (Qiagen, Germantown, MD, USA).

### Exome sequencing and bioinformatics analysis

To identify additional genes for DCM, WES was performed on four members of Family 1(the proband, sibling of the proband, parents of the proband). The normal human population database consisted of The Thousand Genomes Project, ESP6500SI-V2, ExAC Human Exome Integration Database and the sequencing company's internal database. All variants were annotated with ANNOVAR software (version 2014).

### Quantitative RT-PCR

Total RNA was isolated from cells using the Qiagen RNeasy Mini kit. In parallel, we analysed the mRNA concentration of the housekeeping β-actin as an internal control for normalization. The real-time monitoring of the PCR reaction, the precise quantification of the products in the exponential phase of the amplification and the melting curve analysis were performed with the Bio-Rad CFX Manager software, following recommended instructions of the manufacturer.

### Immunocytochemistry

Heart tissue fixed with 4% paraformaldehyde was dehydrated by an automatic dehydrator, embedded, and sectioned as follows. Firstly, the dewaxed sections were placed in 3% methanolic hydrogen peroxide at room temperature for 10 min. Secondly, PBS wash 3 times. Secondly, immersing the sections in 0.01 M citrate buffer (PH 6.0), heating to boiling and disconnecting, after an interval of 5 min, repeated once, after cooling, washed 2 times with PBS. Then, adding normal goat serum blocking solution dropwise. Incubated with primary antibodies at 4 °C overnight. Adding biotinylated secondary antibody dropwise, 37 °C for 30 min. PBS wash 3 times. Mixing the reagents of DAB Color Development Kit, and add dropwise to the sections at room temperature, for about 2 min, then wash with distilled water. Hematoxylin lightly re-stained, dehydrated, transparent, and sealed with neutral gum.

### CRISPR/Cas9 *bicd2* knock out in zebrafish

Zebrafish fertilized eggs were collected. 200 ng/ul Cas9 protein were mixed with ~ 80 ng/ul sgRNAs and subsequently microinjected into zebrafish fertilized eggs (1 nl per embryo). Five embryos were taken when the injected embryos developed to 24 hpf. PCR amplification products were cloned into pGEM-T Easy plasmid (5 μl total volume of reaction). The above 5 μl ligation product was transformed into 50 μl E. coli DH5α receptor cells (pfu ≥ 108). Then coated in LB plates containing ampicillin (50 μg/ml) and incubated upside down overnight at 37 °C. Positive clones were validated by Sanger sequencing.

### Zebrafish echocardiogram

The Vevo2100® Imaging System and Vevo Imaging Station was used to perform transthoracic echocardiography on 7-month-old zebrafish to examine indicators of ventricular function and ventricular size during systole and diastole with a 22–55 MHz transducer probe. Before echocardiography, zebrafish were anesthetized with 0.02% tricaine to induce sedation without cessation of breathing. The two-dimensional B-mode was used for measurement of heart rate, ejection fraction, fractional shortening, end-diastolic and end-systolic area, end-diastolic and end-systolic volume, cardiac output, and stoke volume. The left ventricular volumes are calculated by tracing the endocardial border manually at end diastole and at end systole. B-mode imaging quality was further optimized by adjusting focal depth, gain, image width and depth.

### RNA-seq analysis

Before further data analysis, we firstly checked raw data quality and removed reads with poor quality to get clean reads with high quality. Then all clean reads were mapped to zebrafish genome Assembly GRCz11 using HISAT (version 2.2.1) [23]. StringTie (version 2.2.0) [23] was used to quantify gene expression in different samples and FPKM values were extracted as the expression metric. EBSeq (version 1.34.0) [24] was utilized on raw reads matrix to find out differentially expressed genes. Genes with absolute fold change not less than 2, and adjusted P-value less than 0.05 were defined as differentially expressed genes.

### Statistical analysis

Statistical significance was determined using Student’s t test or Wilcox test. Quantitative data are presented as the means ± SD, as indicated in the figure legends. Statistical analyses were performed using R 4.10.

## Results

### Identification of candidate variants in a consanguineous family with DCM

**Fig. 1:**
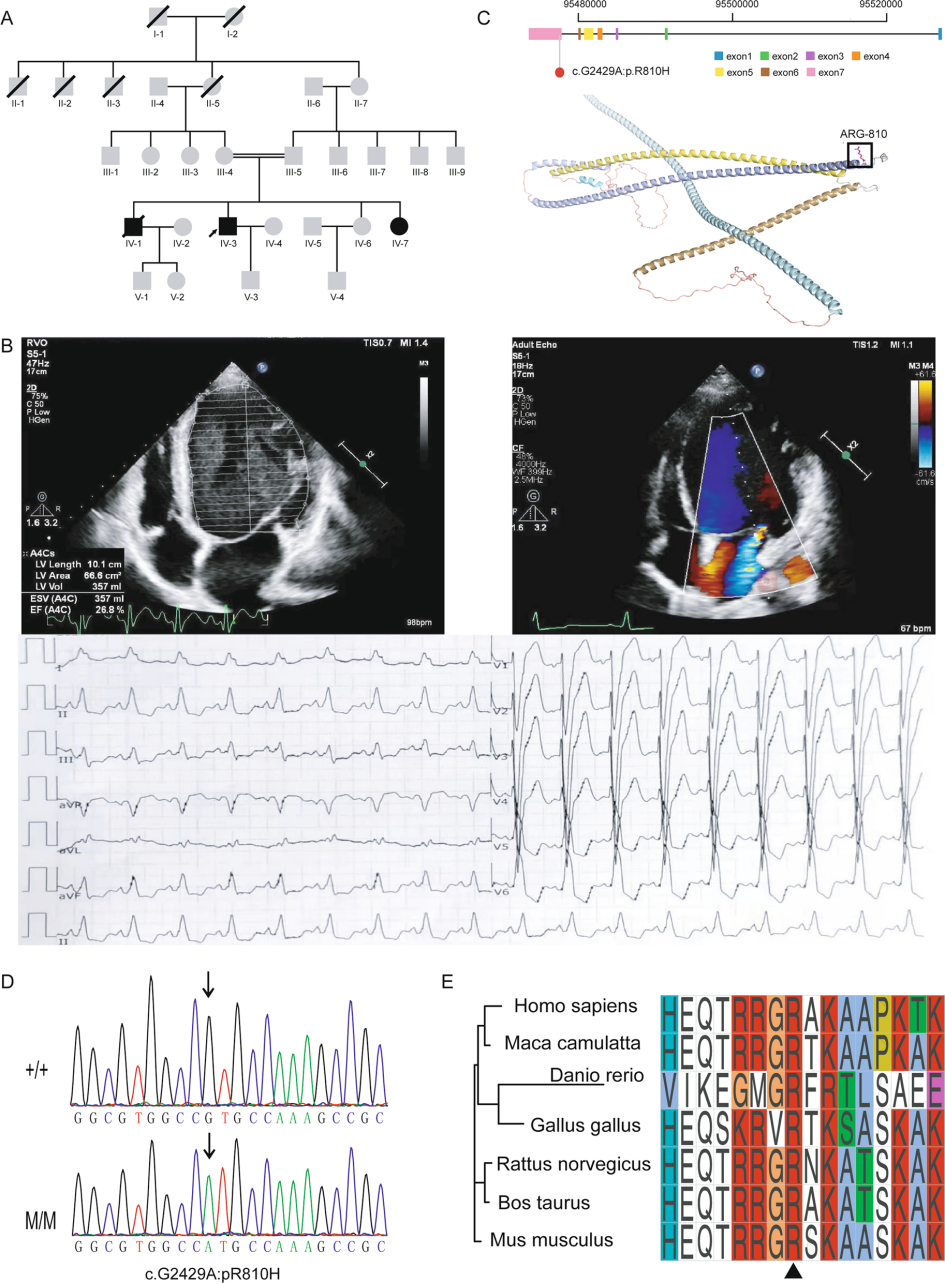
Family pedigree and clinical phenotypes. **A**. Pedigree of the consanguineous family affected by DCM inherited in an autosomal recessive pattern. square represents male and circle represents female. Grey square/circles represent healthy members without DCM. Black square/circles represent DCM patients. The proband is indicated by an arrow. Square/circles with slash sign represent deceased males/females. **B**. Echocardiography and electrocardiogram images of the proband showing dilated heart and decreased LVEF. Upper left panel: parasternal long-axis echocardiogram view of the left ventricle showing two-dimensional measurements of right and left ventricular wall thickness, septal thickness and right and left ventricular internal diameter. Upper right panel: apical four chamber echocardiogram view showing length, area, volume, end-systolic volume (ESV) and ejection fraction of the left ventricle. Lower panel: 12-lead electrocardiogram (ECG) showing paroxysmal sinus tachycardia and complete left bundle branch block. **C**. Candidate DCM variant in *BICD2*. Upper panel: the variant located in exon 7 of *BICD2*. Lower panel: location of the variant in the *BICD2* protein 3D structure predicted by the AlphaFold algorithm. **D**. Sanger sequencing results indicating the genotypes of the family members. Arrows indicate the variant locus. **E**. Protein sequence alignment of the amino acid sequences surrounding the *BICD2* variant with orthologues from *H. sapiens* to *Danio rerio*. Note that the amino acid sequences surrounding the affected amino acid residues are highly conserved

A consanguineous family with 3 DCM-affected members (Family 1) was recruited (Fig. 1A). Conventional and dynamic electrocardiograms suggested occasional first-degree atrioventricular block, paroxysmal sinus tachycardia and complete left bundle branch block (Fig. 1B). The proband was treated with cardiac resynchronization therapy to provide simultaneous electrical activation. The proband’s brother was also DCM patient, and he received a heart transplant. However, the surgery did not substantially reduce his symptom severity, and he died one year later. The proband’s sister was also diagnosed with DCM and she showed frequent atrial premature contractions, and partial of the premature electrical impulse not transmitted downstream to the ventricle. All three DCM patients in the consanguineous family did not show any skeletal muscle abnormalities and their CK serum level was within the normal range. None of the parents or other antecedents showed cardiac dysfunction.

WES was performed on consanguineous family members with DCM but revealed no known DCM-causing gene mutations. We therefore sought new candidate pathogenic variants. WES data was analysed as illustrated (Additional file 1: Fig. S1). In total 14,208 candidate rare variants were obtained. Considering the possible autosomal recessive inheritance pattern, we obtained 56 candidate variants. Finally, 51 variants were excluded based on the American College of Medical Genetics and Genomics 2015 guidelines. The remaining 5 variants in 5 separate genes, *BICD2*, *SERINC1*, *BVES*, *ADCY1* and *PAPPA,* were considered for further evaluation (Additional file 1: Table S1).

*BICD2* is a dynein-activating adaptor protein that functions in minus-end-directed microtubule-based transport by docking dynein motor proteins to appropriate cargos. Reduced *LMNA* protein expression and *MKL-1*/*SRF* activity were observed in *Bicd2*-deficient mice [19]. *LMNA* is a known causative gene for DCM, accounting for 4–8% of patients with DCM [25–27]. In addition, some individuals with spinal muscular atrophy caused by the *BICD2* ‘hot spot’ mutation, c.302C > T:p.Ser107Leu, presented with unexpected heart failure-associated symptoms and exertional and supine dyspnoea [17]. Considering the possible interaction between *LMNA* and *BICD2* and the heart failure symptoms of *BICD2* mutant patients, we hypothesized that the homozygous variant (NM_001003800.1:c.2429G > A) in the C-terminal region of *BICD2* (Fig. 1C) would be a candidate DCM-causative variant in this family.

The missense variant of *BICD2* (Fig. 1C), segregated with the disease phenotype. The Sorting Intolerant From Tolerant [28] algorithm prediction score for the variant was 0.003 (Additional file 1: Fig. S2), indicating a damaging role. Sanger sequencing confirmed that both parents were heterozygous carriers and that the two surviving DCM patients were homozygous at this locus (Fig. 1D). Furthermore, the amino acids affected by this variant are highly evolutionarily conserved in multiple species (Fig. 1E). Together, these findings indicate that the *BICD2* missense variant could be harmful to protein function.

### Three *BICD2* variants in 210 sporadic DCM cases

We further performed Sanger sequencing of *BICD2* in 210 DCM cases and found one missense variant and two synonymous variants (Additional file 1: Fig. S3). The missense variant, located in exon 2 (NM_001003800.1:c.421C > A) (Additional file 1: Fig. S3A), contributed to an amino acid substitution from arginine to serine. Bioinformatic prediction suggested that it was a deleterious variant (Additional file 1: Table S2). The other two variants of *BICD2* were synonymous (Additional file 1: Table S2). These results support the hypothesis that *BICD2* may play a significant role in DCM pathogenesis.

### *BICD2* expression in heart tissue

**Fig. 2:**
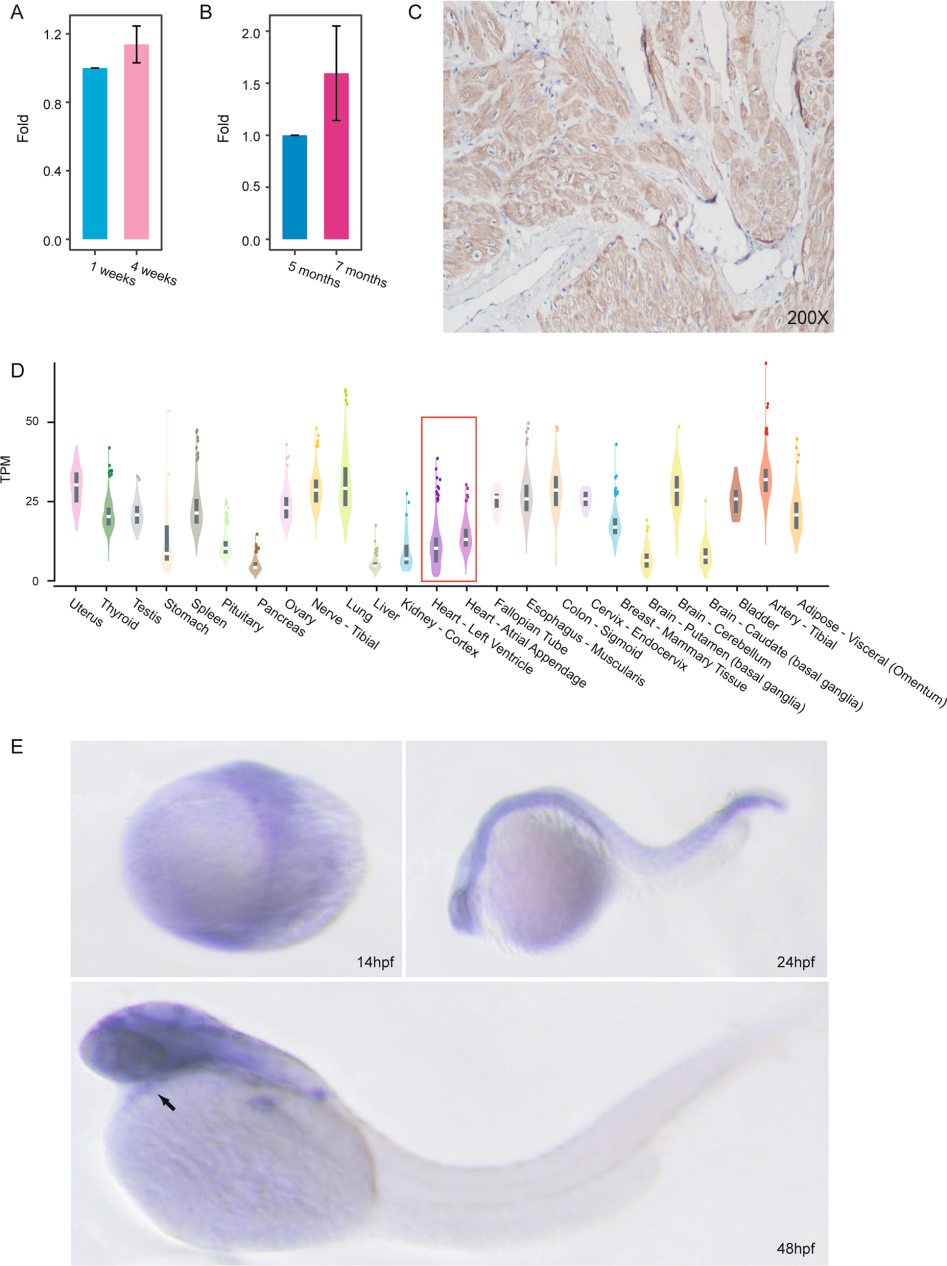
Expression of *BICD2* in human, mouse and zebrafish heart tissues. **A**. qPCR of *Bicd2* in the hearts of C57 mice suggested an increasing trend in expression over the course of development (n = 3). Data are represented as the mean ± SD. **B**. qPCR of *BICD2* in the hearts of zebrafish suggested an increasing trend in expression over the course of development (n = 3). Data are represented as the mean ± SD. **C**. Immunohistochemistry with anti-*BICD2* antibody of normal human heart showing ubiquitous staining. **D**. Bulk RNA-seq revealed the mRNA expression level (transcripts per million fragments) of *BICD2* in multiple tissues from humans. **E**. In situ hybridization of *bicd2* in zebrafish revealed that *bicd2* was expressed in the heart at the embryonic stage

We explored the expression of *BICD2* in heart tissues from humans as well as mice and zebrafish. Real-time PCR results showed that *Bicd2* mRNA expressed in the hearts of mice and that its expression level increased with age (Fig. 2A). A similar expression pattern was also observed in the zebrafish heart, in which the expression of *bicd2* was much higher at 7 months than at 5 months (Fig. 2B).

In addition, immunohistochemical staining of *BICD2* in normal left atrial appendage supported its high expression in human (Fig. 2C). Moreover, RNA-seq of *BICD2* from the GTEx database confirmed its relatively high expression in the human heart (Fig. 2D), and scRNA-seq data of *BICD2* suggested that it was mainly expressed in myocytes, endothelial cells, and fibroblasts (Additional file 1: Fig. S4). Thus, we demonstrated that *BICD2* expressed in the hearts of human, mice and zebrafish at both the mRNA and protein levels.

We further conducted in situ hybridization of *bicd2* in zebrafish at embryonic stages(Fig. 2E). At 48 hpf, *bicd2* was expressed in the retina, forebrain, midbrain and hindbrain regions, with strong expression in the pectoral fin bud base (Fig. 2E). Taken together, these data indicate that *BICD2*/*Bicd2*/*bicd2* are expressed in the heart in multiple species, implying their involvement in heart development and function.

### Knockout of *bicd2* leads to partial embryonic lethality and altered cardiac function in homozygotes

**Fig. 3:**
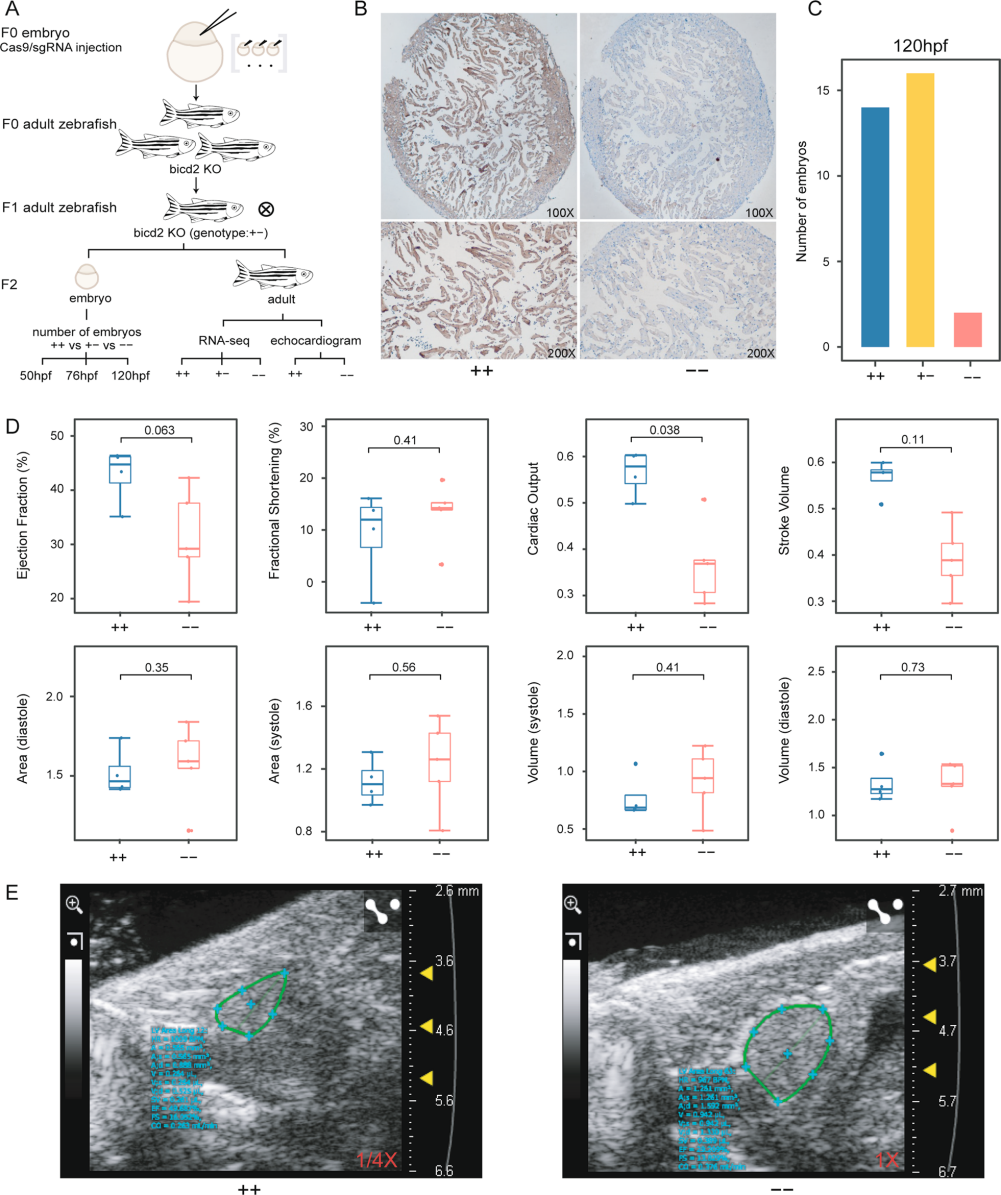
DCM-associated phenotypes in *bicd2*-deficient zebrafish. **A**. Flow chart of *bicd2* zebrafish model development for functional experiments. **B**. Immunohistochemistry of zebrafish hearts with anti-*BICD2* antibody showing the absence of staining in *bicd2*–fish and ubiquitous staining in wild-type fish. **C**. Number of homozygous (n = 2), heterozygous (n = 16) and wild-type (n = 14) embryos at 120 hdf. **D**. Boxplot showing indices of ventricular size, including area, volume, ejection fraction, fractional shortening, cardiac output, and stroke volume, in homozygotes (n = 5) and wild-type fish (n = 4). The lower and upper hinges correspond to the 25th and 75th quartiles. The upper whisker extends from the upper hinge to the largest value no further than 1.5 × IQR from the hinge; The lower whisker extends from the hinge to the smallest value at most 1.5 × IQR of the hinge. Data beyond the end of the whiskers are "outlying" points and are plotted individually. IQR(the inter-quartile range): distance between the first and third quartiles. **E**. Echocardiogram plot showing ejection fraction difference between a homozygous mutant and a wild-type fish

We designed a *bicd2* knockdown assay in zebrafish to unravel the associated phenotypic changes; the experimental workflow is shown in Fig. 3A. We injected Cas9/sgRNA into embryos of F0 generation zebrafish and further screened for *bicd2*-deficient zebrafish in F0 adult zebrafish (Fig. 3A) (CRISPR target sequence in Additional file 1: Table S3). Three fish were obtained and mated with each other to produce *bicd2* heterozygous F1 offspring (Fig. 3A) (PCR primers for F1 mutant identification in Additional file 1: Table S4). Details for the generation of *bicd2*-KO zebrafish are provided in Additional file 1: Fig. S5. We then allowed the F1 generation fish to self-cross to obtain F2 generation fish with three segregating genotypes (Fig. 3A). We measured the survival rate of embryos with different genotypes at three embryonic time points (Fig. 3A). At the adult stage, we performed echocardiographic measurements and transcriptome sequencing to delineate possible regulatory mechanisms(Fig. 3A).

We firstly examined *bicd2* immunostaining to determine whether *bicd2* was efficiently knocked out. Immunostaining images showed lower *bicd2* expression in the *bicd2*–fish (Fig. 3B). Viable embryos’ number was much lower in *bicd2*–than in the other two groups at all three developmental stages (Fig. 3C, Additional file 1: Fig. S6). At 50 hpf, only one *bicd2*–embryo survived (Additional file 1: Fig. S6) (Additional file 1: Table S5), while the number of surviving *bicd2*+−and *bicd2*++embryos were twenty-two and thirteen (Additional file 1: Fig. S6) (Additional file 1: Table S5), respectively. Thus, the proportion of viable homozygous embryos was 2.78%, far lower than the theoretically predicted ratio of 25%. A similar trend was observed at both 76 hpf and 120 hpf (Fig. 3C, Additional file 1: Fig. S6) (Additional file 1: Table S5). The results suggests that *bicd2* plays a vital role in zebrafish embryogenesis and supports the conclusion that bicd2 knockout leads to partial embryonic lethality. The heart rate of *bicd2*–, *bicd2*+−and *bicd2*++fish at three embryonic stages showed no significant difference (Additional file 1: Fig. S7) (Additional file 1: Table S6). In addition, no obvious phenotype was observed during the embryo development period (images not shown).

Electrocardiography was performed on *bicd2*– and *bicd2*++fish at seven months. Ventricular chamber size was assessed based on volume and area. We therefore measured the two-dimensional end-diastolic area (VAd) and end-systolic area (VAs) and three-dimensional end-diastolic volume (EDV) and end-systolic volume (ESV) in both *bicd2*–and *bicd2*++zebrafish. Compared with *bicd2*++zebrafish, *bicd2*–fish showed slightly larger values in both VAd and VAs (Fig. 3D, Additional file 1: Table S7). The EF was much lower in *bicd2*–than in *bicd2*++fish (Wilcox test, *P* value = 0.063) (Fig. 3D, Additional file 1: Table S7). EF < 45% is diagnostic for DCM [29]. In our studies, most *bicd2*–fish presented EF < 45%, supporting that *bicd2* is crucial for cardiac function. Moreover, cardiac output was significantly lower in the *bicd2*– fish than *bicd2*++fish (Wilcox test, *P* value < 0.05) (Fig. 3D, Additional file 1: Table S7). Echocardiographic images representing zebrafish hearts with two different genotypes were extracted from echocardiograms and were compared (Fig. 3E). In summary, these results indicate that *bicd2* knockout may cause abnormal contraction of the heart.

### RNA-seq of bicd2-deficient zebrafish revealed a transcriptomic shift in cardiomyocytes

We conducted transcriptome sequencing of heart tissues from the *bicd2*–, *bicd2*+−and *bicd2*++fish to examine the transcriptional differences. Consistent with expectations, *bicd2* expression was significantly lower in the knockout group than in the *bicd2*++fish (Additional file 1: Table S8). Gene set enrichment analysis (GSEA) [30] based on ranking of all expressed genes can provide more informative biological evidence than enrichment analysis focused solely on differentially expressed genes (DEGs). Therefore, we first utilized GSEA to identify significantly enriched gene sets. Compared to the *bicd2*++fish, the *bicd2*–group showed enrichment of cardiopathy-related signalling pathways and metabolic pathways, for example, KEGG_CARDIAC_MUSCLE_CONTRACTION, KEGG_CALCIUM_SIGNALI-NG_PATHWAY,KEGG_OXIDATIVE_PHOSPHORYLATION,KEGG-GLYCOL

**Fig. 4:**
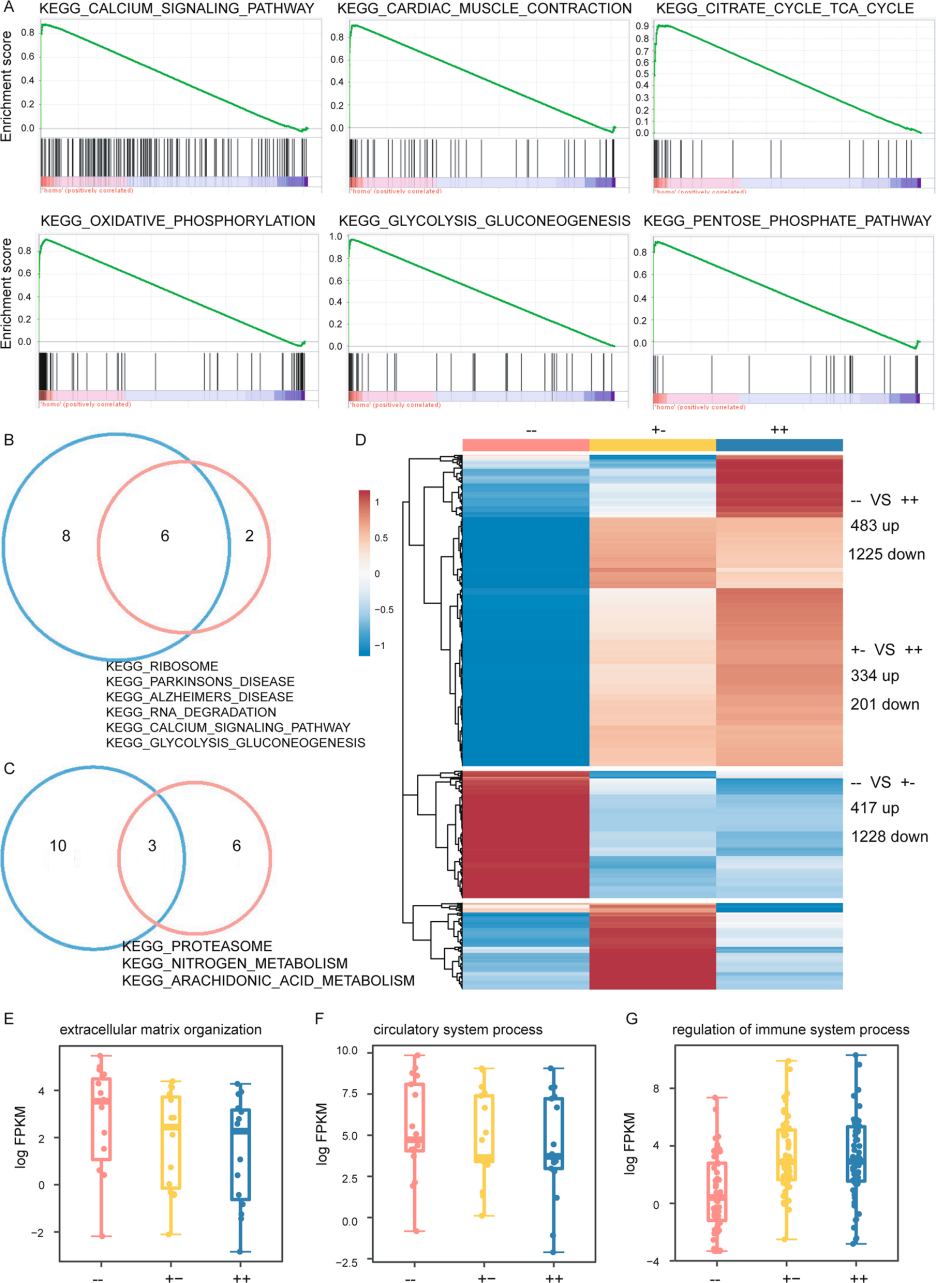
*bicd2*-deficient hearts exhibited cardiac transcriptome shift. **A**. GSEA plots of six KEGG pathways showing higher expression in bicd2 zebrafish. **B**. Venn diagrams showing enriched KEGG pathways among genes with increased expression in the *bicd2*-deficient groups. **C**. Venn diagrams showing enriched KEGG pathways among genes with decreased expression in the *bicd2*-deficient groups. **D**. Differentially expressed genes in the two *bicd2*-deficient groups. Cells are filled according to z score: red indicates higher (activated), blue indicates lower (inhibited). **E**. Differentially expressed genes involved in extracellular matrix organization. Cells are filled according to z score: red indicates higher (activated), blue indicates lower (inhibited). **F**. Differentially expressed genes involved in circulatory system processes. Cells are filled according to z score: red indicates higher (activated), blue indicates lower (inhibited). **G**. Differentially expressed genes involved in the regulation of immune system processes. Cells are filled according to z score: red indicates higher (activated), blue indicates lower (inhibited)

YSIS_GLUCONEOGENESIS, KEGG_CITRATE_CYCLE_TCA_CYCLE and KEGG_PENTOSE_PHOSPHATE_PATHWAY (Fig. 4A), as well as pathways associated with nervous system disease (Additional file 1: Table S9). These results are consistent with functional explorations of *Bicd2* in mice, which proved a causal role of *Bicd2* in neuronal disorders [19]. In addition, pathways related to mitochondrial energy metabolism, such as oxidative phosphorylation (OXPH) and the tricarboxylic acid cycle (TCA), are common characteristics of distinct heart failure, as demonstrated previously [31, 32]. Interestingly, 6 out of 14 pathways (Fig. 4B) exhibited increases in expression in both the *bicd2*–and *bicd2*+−fish, including KEGG_CALCIUM_SIGNALING_PATHWAY and KEGG_GLYCOLYSIS_GLU.

-CONEOGENESIS. Only 3 common pathways exhibited decreased expression in those two groups (Fig. 4C), implying the transcriptome differences between the *bicd2*–and *bicd2*+−fish.

We then extracted DEGs to further delineate biologically meaningful pathways involved in the *bicd2* gene regulatory network. As expected, the *bicd2*–fish displayed the largest change among all three groups, with a total of 1708 DEGs (483 upregulated genes, 1225 downregulated genes, Fig. 4D, Additional file 2: Table S10). Surprisingly, the difference between the two groups of *bicd2*-KO genotypes was much larger than that between the *bicd2*+−fish and the *bicd2*++fish. In detail, there were 1645 DEGs (417 upregulated genes, 1228 downregulated genes, Fig. 4D, Additional file 3: Table S11) between the *bicd2–*fish and the *bicd2*+−fish*.* And 535 DEGs (334 upregulated genes, 201 downregulated genes, Fig. 4D, Additional file 4: Table S12) between the *bicd2*+−fish and the *bicd2*++fish. The top 15 enriched GO pathways based on 1708 DEGs discovered between the *bicd2*–fish and the *bicd2*++fish are displayed (Additional file 1: Fig. S8-9). Genes with increased expression in the *bicd2*++fish were mainly related to blood vessel development, extracellular matrix organization, cell–cell adhesion, carbohydrate metabolic process and circulatory system process (Additional file 1: Fig. S8). However, the genes with decreased expression were mainly related to the immune system, including regulation of immune system processes, response to biotic stimulus, and leukocyte activation (Additional file 1: Fig. S9). Notably, an increased expression of genes encoding extracellular matrix proteins in DCM patients was previously demonstrated by microarray [31]. In our study, extracellular matrix genes (Fig. 4E) (Additional file 1: Fig. S10), such as *col4a2*, *col4a4*, *col5a1*, *col8a1b*, *col18a1b*, and genes involved in circulatory system processes showed higher expression in the homozygous group than in the wild-type group (Fig. 4F) (Additional file 1: Fig. 11). Genes related to the regulation of immune system processes (Fig. 4G) (Additional file 1: Fig. 12), such as *cd74b* and *cd79a,* exhibited decreased expression in the two *bicd2*-KO groups, implying activation of the immune system after *bicd2* knockout in zebrafish.

A set of 51 genes with evidence of association with susceptibility to inheritable DCM in humans was established in a previous study [4]. Orthologue retrieval in the zfin data report and Ensembl identified zebrafish homologues for 48 of these genes (Additional file 5: Table S13). Among these 48 genes, 28 had a single orthologue in zebrafish (Additional file 1: Fig. S13, Additional file 5: Table S13). 8 out of these 28 orthologues, including *bag3*, *nebl*, *psen2*, *jph2*, *dtna*, *psen1*, *gatad1* and *plekhm2,* showed the lowest expression in the homozygous group (Additional file 1: Fig. S13). In contrast, 10 out of 28 orthologues, including *mybpc3*, *lama4*, *nppa*, *tnni3k*, *cmlc1*, *nexn*, *lrrc10*, *csrp3*, *abcc9*, and *tcap,* showed the highest expression in the homozygous group (Additional file 1: Fig. S13). Multiple zebrafish orthologues were identified for 20 human DCM candidate genes (Additional file 1: Fig. S14).

## Discussion

Most previously reported familial DCM cases are caused by variants inherited in an autosomal dominant pattern [3]. Currently, more than 250 variant genes spanning > 10 gene ontologies have been suggested to contribute to inherited DCM [4]. However, the heritability of DCM cannot be completely explained by the variants discovered thus far. Here, we searched for the pathological variant causing DCM in a consanguineous family and discovered *BICD2* as a novel DCM candidate disease gene. Notably, the inheritance mode of DCM in the consanguineous family was autosomal recessive, suggesting that loss of function contributes to the disease.

*BICD2* is a dynein-activating adaptor protein that was previously implicated as a causative gene in autosomal dominant spinal muscular atrophy. Individuals carrying heterozygous missense variants in BICD2 exhibit muscle weakness and atrophy predominantly of the proximal lower limbs. However, the inheritance mode of BICD2 in our DCM family was autosomal recessive. None of the previous DCM genetic researches have reported functional role of BICD2 or the association of BICD2 to DCM. Our functional characterization of BICD2 in DCM may advance our understanding of the genetic underpinnings of DCM, facilitating early genetic screening of familial DCM.

However, a limitation of our work is that we have not yet proven a direct relationship between the *BICD2* variant identified and phenotypes associated with an enlarged heart. We will continue to explore the cardiac dysfunction caused by this *BICD2* variant. We will continue our work in elucidating molecular mechanism underlying the pathogenesis of familial DCM.

## Conclusions

Our study shows that *BICD2*, an adapter protein linking the dynein motor complex to various cargos, is a novel DCM candidate gene and conceptually expands our horizons regarding pathogenesis of DCM.

## Supplementary Information

**Additional file 1**. Identification of candidate disease gene for the consanguineous family with dilated cardiomyopathy.

**Additional file 2**. Differentially expressed genes in homozygous versus wild-type zebrafish.

**Additional file 3**. Differentially expressed genes in heterozygous versus homozygous zebrafish.

**Additional file 4**. Differentially expressed genes in heterozygous versus wild-type zebrafish.

**Additional file 5**. Zebrafish homologues of 51 human DCM-associated genes.

## Data Availability

The datasets are available on China National GeneBank DataBase(CNGBdb) under project accession CNP0003349 (https://db.cngb.org/cnsa/variant/CNP0003349_2f12e5db/reviewlink/).
