# Supplementary material for: Identification and functional characterization of BICD2 as a candidate disease gene in an consanguineous family with dilated cardiomyopathy
Source: BMC Med Genomics. 2022 Sep 6;15:189. doi: 10.1186/s12920-022-01349-y (PMC9446846; doi:10.1186/s12920-022-01349-y)
Supplement: Supplementary file 1 — Additional file 1. Identification of candidate disease gene for the consanguineous family with dilated cardiomyopathy. [file 12920_2022_1349_MOESM1_ESM.pdf]

Supplementary Figures

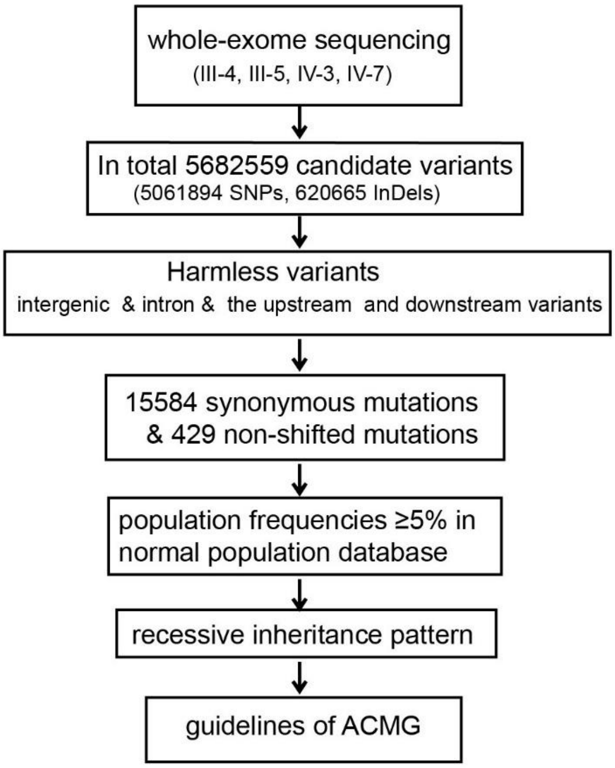

Supplementary Figure 1. Workflow of whole-exome sequencing analysis for the consanguineous family with dilated cardiomyopathy.

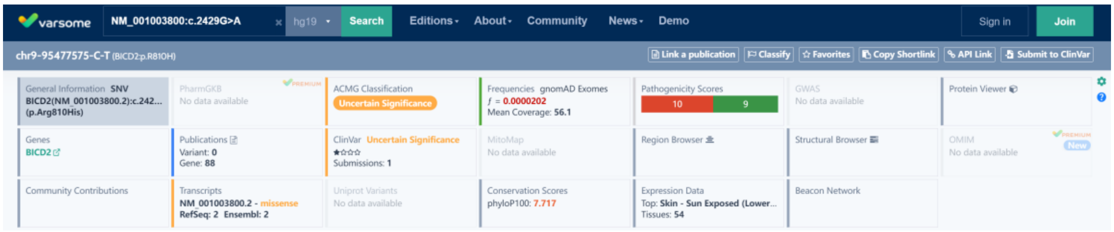

Supplementary Figure 2. Algorithm prediction for the *BICD2* missense variant (c.2429G>A:p.Arg810His) identified in DCM patients from the consanguineous family.

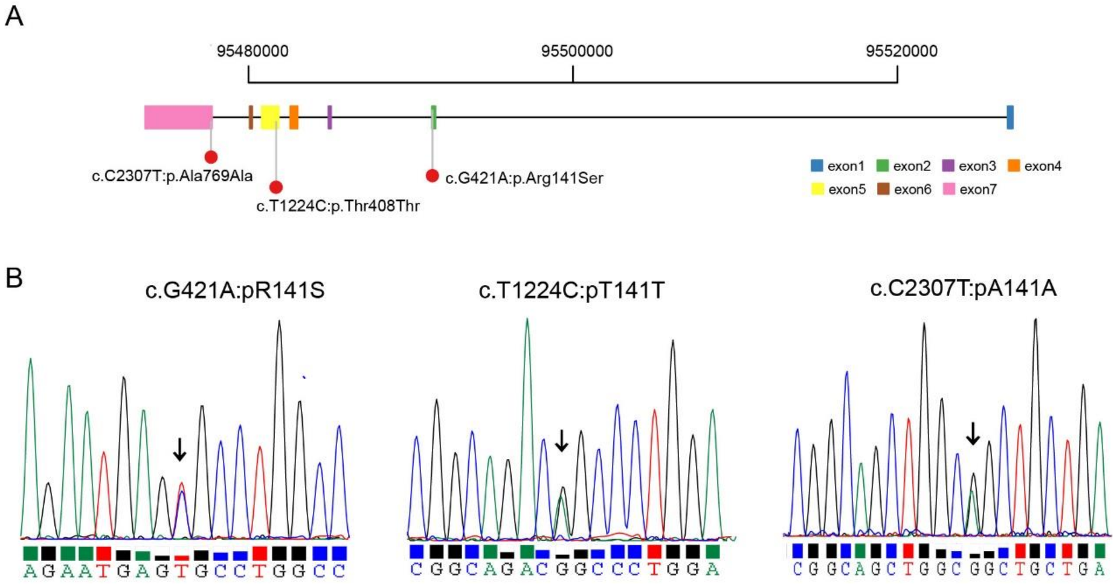

Supplementary Figure 3. Validation of *BICD2* in 210 sporadic DCM patients.

A. Three *BICD2* variants were identified in 210 sporadic DCM patients.

B. Sanger sequencing results of the missense variant.

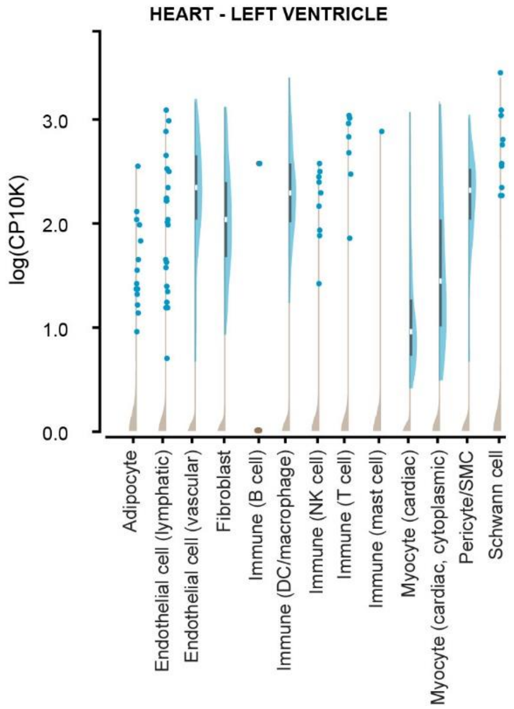

Supplementary Figure 4. scRNA-seq of the human heart unraveled *BICD2* mRNA expression in different cell types.

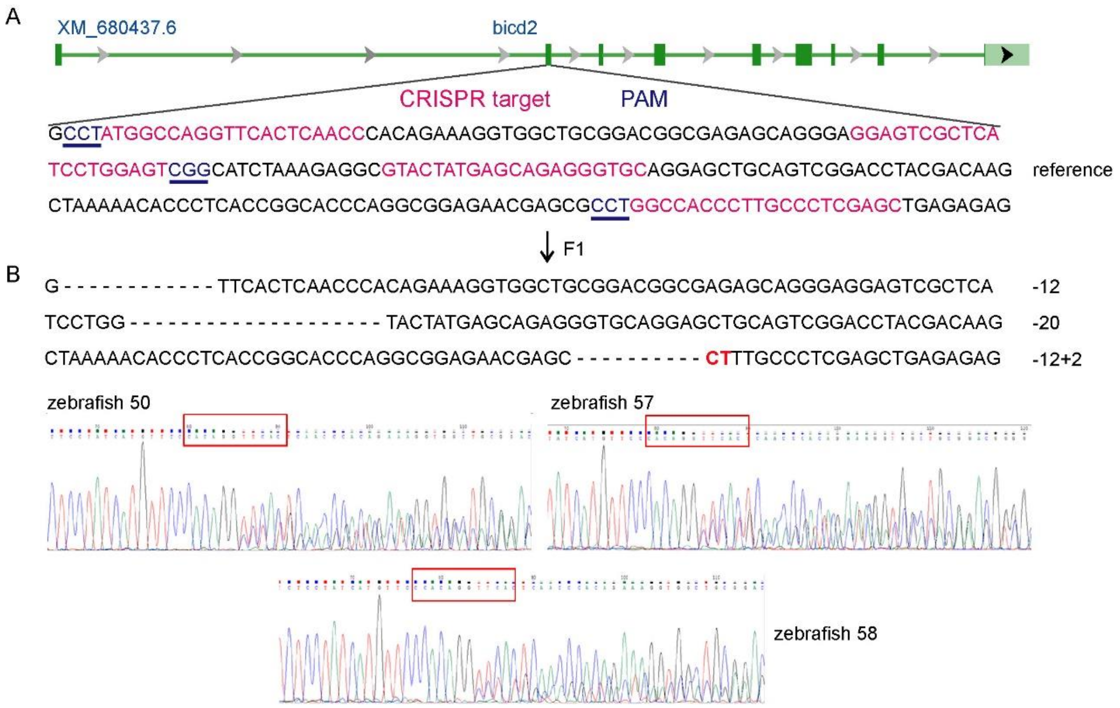

Supplementary Figure 5. Generation of bicd2 knockout zebrafish.

A. The target site of the CRISPR/Cas9 system, which was designed at the exon 2 of the bicd2 gene, consists of the red colored CRISPR target sequence and the CCT/CGG/CCT protospacer-adjacent sequence. We identified 3 heterozygous mutant adult zebrafish.

B. Sequencing validation of the F1 heterozygous mutant adult zebrafish (3 fish with the same mutant genotype).

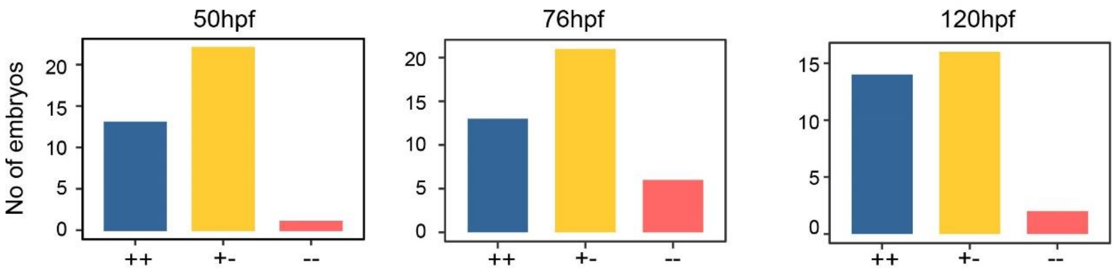

Supplementary Figure 6. Number of embryos in three different zebrafish groups with genotype of wild-type, heterozygous and homozygous

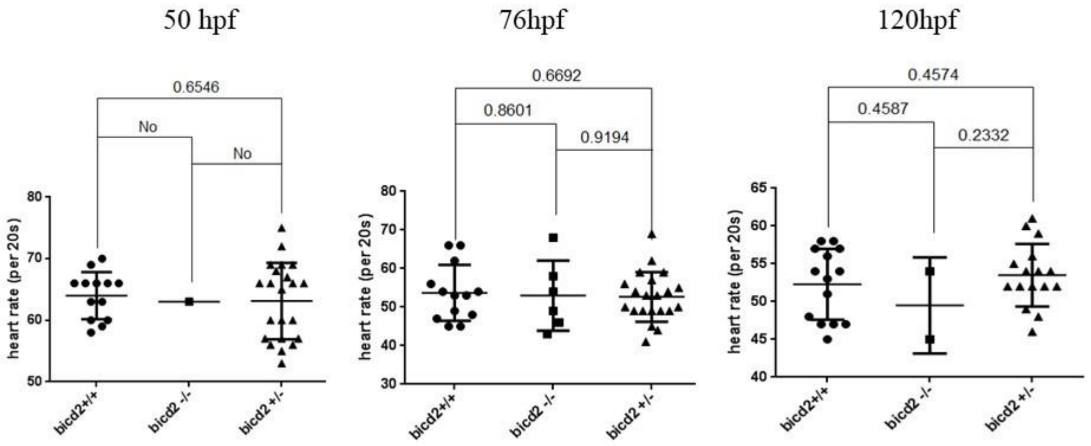

Supplementary Figure 7. Heart rate of embryo zebrafish in three different zebrafish groups with genotype of wild-type, homozygous and heterozygous.

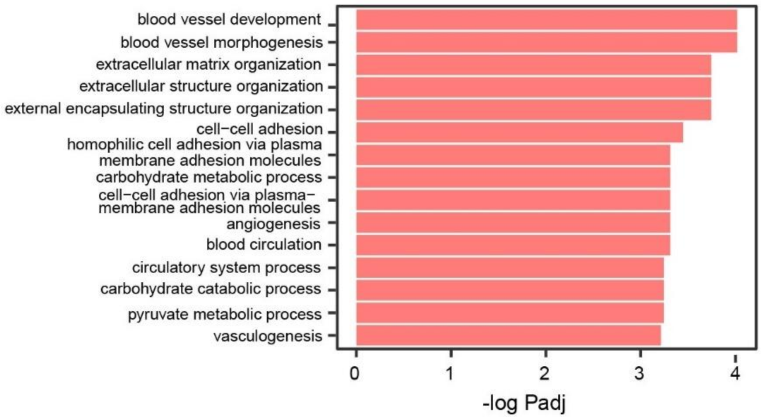

Supplementary Figure 8. The top 15 GO biological pathway increased in *bicd2* homozygous group.

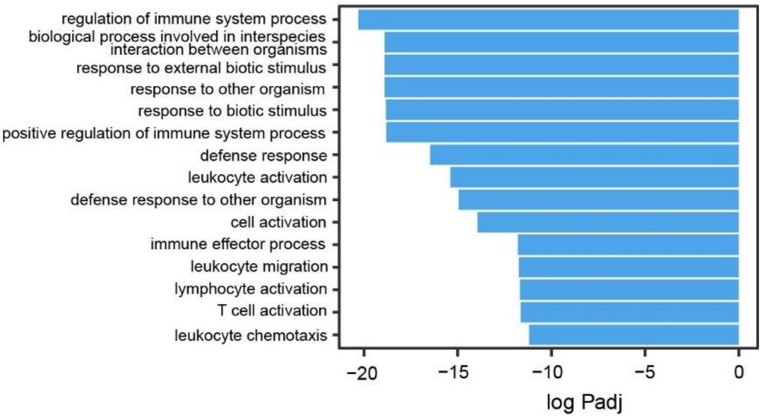

Supplementary Figure 9. The top 15 GO biological pathway decreased in *bicd2* homozygous group.

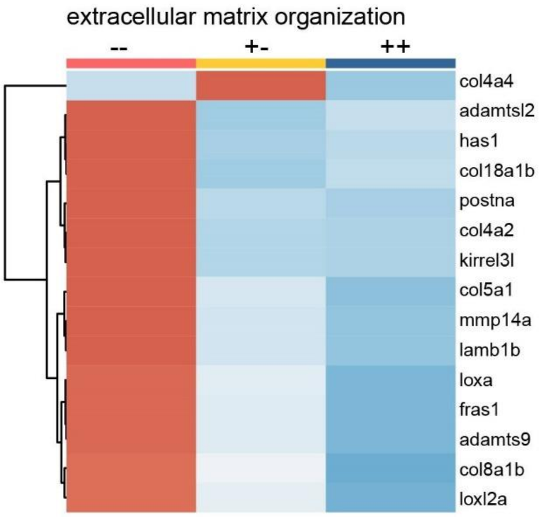

Supplementary Figure 10. Differential expressed genes in extracellular matrix organization. -- represents fishes with bicd2 homozygous genotype, +- represents fishes with bicd2 heterozygous genotype, ++ represents wild-type fish group.

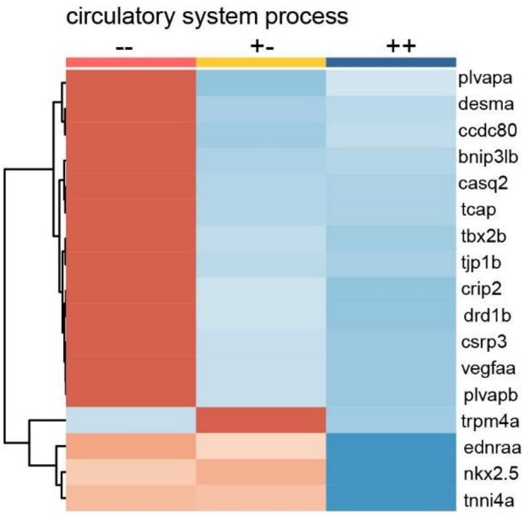

Supplementary Figure 11. Differential expressed genes in circulatory system process. -- represents fishes with bicd2 homozygous genotype, +- represents fishes with bicd2 heterozygous genotype, ++ represents wild-type fish group.

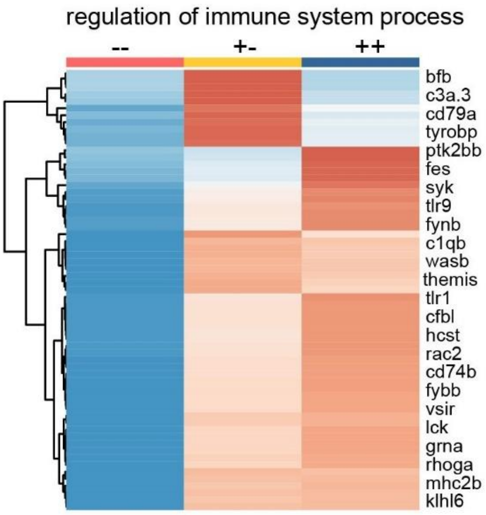

Supplementary Figure 12. Differential expressed genes in regulation of immune system process. -- represents fishes with bicd2 homozygous genotype, +- represents fishes with bicd2 heterozygous genotype, ++ represents wild-type fish group.

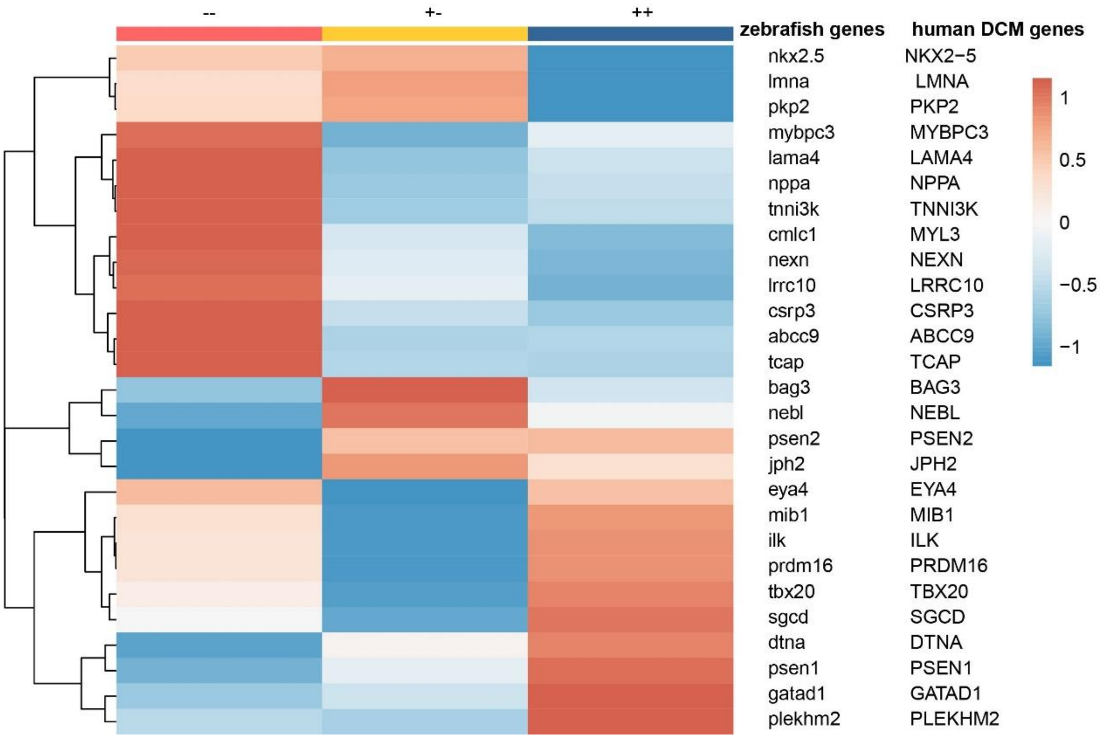

Supplementary Figure 13. Expression of zebrafish homologues of 20 human DCM associated genes with unique homologues in *bicd2*-deficiency zebrafish. -- represents fishes with bicd2 homozygous genotype, +- represents fishes with bicd2 heterozygous genotype, ++

represents wild-type fish group.

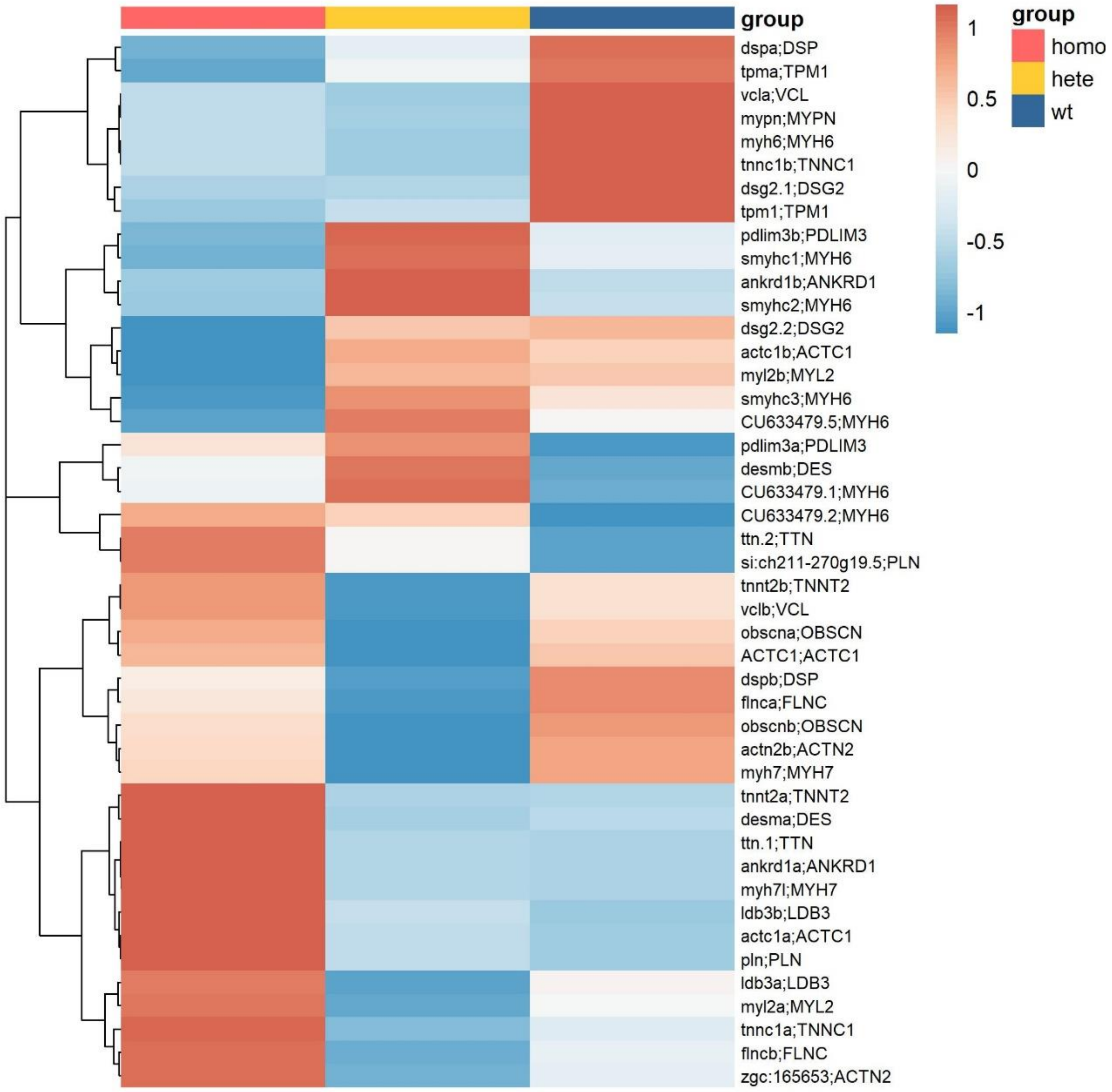

Supplementary Figure 14. Expression of zebrafish homologues of 20 human DCM associated genes with multiple homologues in bicd2-deficiency zebrafish.

Supplementary Table 1. Five candidate DCM disease genes for the consanguineous family with dilated cardiomyopathy.

CHR	POS	REF	ALT	Impact_score	Gene_symbol	High_effect	Hgv.p	Hgv.c	Annotation_Impact
6	105573356	CA	C	0.823213843	BVES	frameshift_variant	p.Cys150fs	c.448delT	HIGH
6	122773161	C	T	0.737171748	SERINC1	missense_variant	p.Val211Ile	c.631G>A	MODERATE
7	45717651	C	T	0.82589953	ADCY1	missense_variant	p.Arg597Trp	c.1789C>T	MODERATE
9	95477575	C	T	0.853430607	BICD2	missense_variant	p.Arg810His	c.2429G>A	MODERATE
9	118997457	G	A	0.83169857	PAPPA	missense_variant	p.Arg758His	c.2273G>A	MODERATE

Supplementary Table 2. Three BICD2 variants identified in 210 sporadic DCM cases

Markers	Exon	Amino acid change	Polyphen2_HDIV_ score	Polyphen2_HDIV_ pred
NM_001003800.1:c.421C>A	exon2	p.Arg141Ser	0.959	D
NM_001003800.1:c.1224A>C	exon5	p.Thr408Thr	-	-
NM_001003800.1:c2307G>T	exon7	p.Ala769Ala	-	-

Supplementary Table 3. CRISPR target sequence

ID	sequence
Guide #1	GGTTGAGTGAACCTGGCCATAGG
Guide #2	GGAGTCGCTCATCCTGGAGTCGG
Guide #3	GTACTATGAGCAGAGGGTGCAGG
Guide #4	GCTCGAGGGCAAGGGTGGCCAGG

Supplementary Table 4. PCR primer for F1 mutant identification

ID	sequence
F1-bicd2	TAAACTTCAAACCTCCACCCCC
R1-bicd2	AGCTTAACATTTCATTGGAGGAGC
F2-bicd2	TCCTCCACCCTACAAGACAAG
R2-bicd2	CGATCCACTGGCTTTAGTTTG

Supplementary Table 5. Number of zebrafish embryos in three genotypes groups

	50hpf	76hpf	120hpf
bicd2+/-	22	21	16
bicd2+/+	13	13	14
bicd2-/-	1	6	2

Supplementary Table 6. Heart rate of zebrafish with three different genotypes at embryo stage

50hpf				76hpf				120hdf			
ID	*bicd2* ++	*bicd2* --	*bicd2* +-	ID	*bicd* ++	*bicd2* --	*bicd2* +-	ID	*bicd2* ++	*bicd2* --	*bicd2* +-
1	70	63	56	1	45	46	49	1	47	54	46
2	66	57	-	2	45	43	54	2	48	45	52
3	60	69	-	3	49	58	49	3	54	52	-
4	59	66	-	4	53	49	45	4	47	48	-
5	69	75	-	5	48	54	41	5	58	56	-
6	63	57	-	6	54	68	50	6	47	52	-
7	66	65	-	7	56	49	-	7	56	52	-
8	58	66	-	8	62	44	-	8	45	59	-
9	63	60	-	9	53	54	-	9	51	60	-
10	66	67	-	10	54	55	-	10	57	54	-
11	60	66	-	11	47	49	-	11	54	52	-
12	66	69	-	12	66	50	-	12	53	49	-
13	66	56	-	13	66	56	-	13	57	61	-
14	69	-	-	14	49	-	-	14	58	55	-
15	53	-	-	15	59	-	-	15	54	-	-
16	55	-	-	16	53	-	-	16	54	-	-
17	60	-	-	17	53	-	-				
18	68	-	-	18	57	-	-				
19	57	-	-	19	69	-	-				
20	66	-	-								
21	60	-	-								
22	72	-	-								

Supplementary Table 7. Metrics of cardiac function and cardiac size in zebrafish groups

	Homo1	Homo2	Homo3	Homo4	Homo5	WT1	WT2	WT3	WT4
Area	1.1201	1.4286	0.8070	1.2610	1.5397	0.9706	1.3079	1.1502	1.0556
Area;s	1.1201	1.4286	0.8070	1.2610	1.5397	0.9706	1.3079	1.1502	1.0556
Area;d	1.5488	1.7202	1.1557	1.5917	1.8398	1.4308	1.7386	1.5010	1.4161
Volume	0.8153	1.1094	0.4857	0.9418	1.2233	0.6637	1.0667	0.6690	0.7015
Volume;s	0.8153	1.1094	0.4857	0.9418	1.2233	0.6637	1.0667	0.6690	0.7015
Volume;d	1.3071	1.5346	0.8416	1.3304	1.5187	1.1730	1.6444	1.2477	1.3011
Stroke Volume	0.4917	0.4253	0.3559	0.3886	0.2953	0.5093	0.5778	0.5787	0.5996
Ejection Fraction	37.6203	27.7109	42.2908	29.2090	19.4459	43.4167	35.1350	46.3777	46.0841
Fractional Shortening	14.7300	3.9495	18.7594	13.8289	13.5571	15.5385	13.4224	-2.7793	10.1920
Cardiac Output	0.5069	0.2833	0.3687	0.3758	0.3066	0.4979	0.5560	0.6009	0.6031

Supplementary Table 8. Mean FPKM of *bicd2* in three zebrafish groups

	homo	hete	wt
bicd2	3.271738	3.949286	5.322773

Supplementary Table 9. KEGG pathways enriched in *bicd2*-- zebrafish

NAME	SIZE	ES	NES	NOM p-val	FDR q-val	FWER p-val	RANK AT MAX	LEADING EDGE
KEGG_PARKINSONS_DISEASE	106.000	0.948	1.720	0.016	0.168	0.170	404.000	tags=58%, list=3%, signal=60%
KEGG_HUNTINGTONS_DISEASE	156.000	0.943	1.703	0.014	0.225	0.389	404.000	tags=39%, list=3%, signal=40%
KEGG_GLYCOLYSIS_GLUCONEOGENESIS	53.000	0.970	1.677	0.016	0.373	0.707	204.000	tags=32%, list=1%, signal=32%
KEGG_ALZHEIMERS_DISEASE	135.000	0.915	1.643	0.042	0.532	0.901	404.000	tags=44%, list=3%, signal=45%
KEGG_OXIDATIVE_PHOSPHORYLATION	104.000	0.906	1.628	0.055	0.520	0.939	404.000	tags=57%, list=3%, signal=58%
KEGG_CARDIAC_MUSCLE_CONTRACTION	66.000	0.910	1.610	0.065	0.524	0.966	343.000	tags=32%, list=2%, signal=32%
KEGG_CALCIUM_SIGNALING_PATHWAY	154.000	0.878	1.576	0.082	0.573	0.986	274.000	tags=4%, list=2%, signal=4%
KEGG_FRUCTOSE_AND_MANNOSE_METABOLISM	31.000	0.925	1.571	0.056	0.514	0.987	248.000	tags=13%, list=2%, signal=13%
KEGG_PYRUVATE_METABOLISM	34.000	0.925	1.563	0.055	0.477	0.990	486.000	tags=38%, list=3%, signal=39%
KEGG_CITRATE_CYCLE_TCA_CYCLE	29.000	0.914	1.545	0.061	0.471	0.994	277.000	tags=31%, list=2%, signal=32%
KEGG_RNA_DEGRADATION	54.000	0.871	1.513	0.085	0.499	0.998	10.000	tags=2%, list=0%, signal=2%
KEGG_RIBOSOME	76.000	0.838	1.498	0.096	0.489	0.998	494.000	tags=70%, list=3%, signal=72%
KEGG_PENTOSE_PHOSPHATE_PATHWAY	23.000	0.893	1.473	0.085	0.503	1.000	248.000	tags=17%, list=2%, signal=18%
KEGG_PROPANOATE_METABOLISM	28.000	0.872	1.464	0.088	0.487	1.000	575.000	tags=29%, list=4%, signal=30%
